# Sequential Oxidation Strategy for the Fabrication of Liquid Metal Electrothermal Thin Film with Desired Printing and Functional Property

**DOI:** 10.3390/mi12121539

**Published:** 2021-12-10

**Authors:** Jun-Heng Fu, Xu-Dong Zhang, Peng Qin, Jing Liu

**Affiliations:** 1 Key Laboratory of Cryogenics, Technical Institute of Physics and Chemistry, Chinese Academy of Sciences, Beijing 100190, China; fujunheng17@mails.ucas.ac.cn (J.-H.F.); qinpeng17@mails.ucas.ac.cn (P.Q.); 2 School of Future Technology, University of Chinese Academy of Sciences, Beijing 100049, China; 3 Beijing Key Laboratory of Cryo-Biomedical Engineering, Beijing 100190, China; 4 Key Laboratory of Thermal Science and Power Engineering of Ministry of Education, Department of Engineering Mechanics, Tsinghua University, Beijing 100084, China; 18811345210@163.com; 5 Department of Biomedical Engineering, School of Medicine, Tsinghua University, Beijing 100084, China

**Keywords:** electrothermal film, liquid metal, oxidizing adhesion, composite materials, energy devices

## Abstract

Room temperature liquid metal (LM) showcases a great promise in the fields of flexible functional thin film due to its favorable characteristics of flexibility, inherent conductivity, and printability. Current fabrication strategies of liquid metal film are substrate structure specific and sustain from unanticipated smearing effects. Herein, this paper reported a facile fabrication of liquid metal composite film via sequentially regulating oxidation to change the adhesion characteristics, targeting the ability of electrical connection and electrothermal conversion. The composite film was then made of the electrically resistive layer (oxidizing liquid metal) and the insulating Polyimide film (PI film) substrate, which has the advantages of electrical insulation and ultra-wide temperature working range, and its thickness is only 50 μm. The electrical resistance of composite film can maintain constant for 6 h and could work normally. Additionally, the heating film exhibited excellent thermal switching characteristics that can reach temperature equilibrium within 100 s, and recovery to ambient temperature within 50 s. The maximum working temperature of the as-prepared film is 115 °C, which is consistent with the result of the theoretical calculation, demonstrating a good electrothermal conversion capability. Finally, the heating application under extreme low temperature (−196 °C) was achieved. This conceptual study showed the promising value of the prototype strategy to the specific application areas such as the field of smart homes, flexible electronics, wearable thermal management, and high-performance heating systems.

## 1. Introduction

Gallium-based liquid metals, which own excellent conductivity and rheological properties, can commendably achieve the functions of flexible electronics [1,2,3,4], energy and power transport [5,6], space exploration [7], and tumor therapy [8]. Moreover, the cost-effective preparation strategies, such as printings, are a particular advantage of liquid metal electronics [2,3,4,5,6,7,8,9,10]. The thermal characteristics of metallic material play a vital role in these applications. However, due to the large surface tension of liquid metal (Ga, ~0.7 N/m) [6], it is a practical bottleneck to perform arbitrary printing processes on the flexible substrate. The universally diverse strategies to increase the adhesion between liquid metal and the substrate are explored, such as mechanical microstructure [11], surface coating modification [12,13], and additive doping [14,15,16].

Oxidation offers a cost-effective avenue for the improvement of the interfacial adhesion and chemical activity of liquid metal [17,18,19]. The wettability of the liquid metal on the diverse substrates can be effectively altered by adjusting the thickness and constituent of the oxide layer, and the thickness of the layer will also significantly change the conductive properties of the liquid metal [17,18,19,20,21,22]. However, the current attractive attentions are in the self-limiting and chemical properties of the natural oxide layer on the surface of liquid metal, and there are few reports on the adhesion properties after sufficient oxidation [23,24].

The electrothermal effect is an energy conversion process that converts electrical energy into thermal energy, which has many potential applications in aerospace, biomedical, and industrial applications [25,26]. The heating devices prepared by the traditional metal wire have the problems of local overheating and low flexibility. The reduction in the thickness of sensitive components would increase the risk of function failure. In recent years, flexible heating devices incorporating with graphene and carbon nanotube materials have been widely investigated to replace the commercial rigid electrical heating films [27,28,29,30,31]. However, there are also some limitations on the heating voltage, the achievable maximum heating temperature, and cost-efficiency of such emerging materials, compared with commercial metallic wire film.

Due to its excellent electrical insulation and high temperature resistance, polyimide films (PI) are widely used as thermal control coatings and flexible expandable substrates [32,33,34,35]. Additionally, the flexible electrothermal PI film can normally work in extreme environments, such as aerospace heating, in which the existing electric heating materials are mostly copper–nickel (Cu/Ni) foil or wire as electric heating layer [33,34,35,36]. However, the adhesion between the filament or foil material and the film substrate is connected by a physical squeeze, which is likely to cause local thermal stress or disconnection, thus resulting in a device failure.

Herein, a universal and facile approach to fabricate the liquid metal composite circuit was provided through the oxidizing strategy, in which commercial PI film with excellent electrical insulation was used as the substrate [34,35]. We firstly obtained the surface morphology and oxygen content of the liquid metal with different stirring times, as well as the wetting behavior on the diverse flexible substrates. The electrical performance of liquid metal under various bending angles and long-term power-loading conditions were evaluated. Then, the surface morphologies and electromechanical and electrothermal performances of the prepared film were measured. Finally, the proof-of-concept heating device was applied to heat transfer and demonstrated its stable performance after treatment at extreme temperatures (−196 °C). The flexible heating device proposed in this paper provides a new strategy for flexible heating applications and flexible electronics.

## 2. Materials and Methods

The liquid metal (LM, GaIn_20_Sn_12_) is composed of 68% gallium, 20% indium, and 12% tin by weight, whose melting point is 10.8 °C. Here, GaIn_20_Sn_12_ was stirred at a speed of 650 rpm for 0, 10, 30, 60, 120, or 180 min to obtain oxidizing liquid metal (LM-O). LM-O_0_ represented the striation of liquid metal for 0 min, and LM-O_10_, LM-O_30_, LM-O_60_, LM-O_120_, and LM-O_180_ was the mixture after stirring for 10 min, 30 min, 60 min, 120 min, and 180 min, respectively, with same speed of 650 rpm. Additionally, the commercial PI film was purchased from local stores (BoXu hardware Store, Beijing, China). To insure the glossy and unoxidized feature, LM-O_0_ was treated with alkaline solution (NaOH, 0.1 M). the LM-O_60_ as conductive layer was printed on the copper foil, PET sheet, cellulose paper, and PI film to obtain the heating film through the Chinese brush.

The oxide morphologies of liquid metal and the microstructure of the as-prepared film were acquired with environmental scanning electron microscope (ESEM, QUANTA FEG 250, Hillsboro, OR, USA). The elements’ distribution and mass weight of oxygen were measured with energy disperse spectroscopy (EDS, X-Max 80, Oxford Instruments, London, UK), and X-ray photoelectron spectroscopy (XPS, ESCALAB 250 Xi, Thermo Fisher Scientific Co., Ltd. Hillsboro, OR, USA) was applied to analyzing the valence states of liquid metal. The thermal stability was evaluated by thermogravimetric analysis (TGA, STA 449C NETZSCH, Selb, Germany). Atomic force microscopy (AFM, Fastscan IconBio, Bruker, MA, USA) was applied to characterize the surface roughness of the prepared film. The electrical resistance of the oxidizing liquid metal and the prepared composite film were obtained by digital multimeter via four-probe measurement (Agilent 34420A/34970, Agilent, PaloAlto, CA, USA). The pull–push testing of adhesion force was performed by surface tension meter (DCA21, Dataphysics, Filderstadt, Germany), and the infrared information was recorded by the far-infrared thermograph imaging system (FLIR SC620, FLIR Systems Inc., Wilsonville, OR, USA).

## 3. Results

### 3.1. The Characteristics of the Oxidizing Liquid Metal

**Figure 1:**
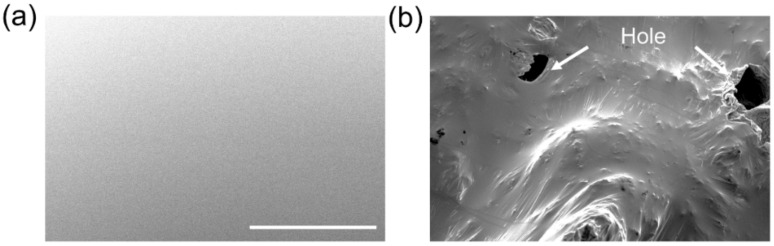
The morphology of (**a**) the pure liquid metal and (**b**) oxidizing liquid metal, and the scale bar is 100 μm.

Gallium-based liquid metals can be fully oxidized via continuously stirring in the ambient atmosphere. Figure 1 showcases the surface morphology of pure liquid metal and oxidized liquid metal, in which the surface of pure LM was smooth and homogeneous. Due to the tunneling effect, the electrons in the liquid metal inner core would be transferred to the surface of the absorbed oxygen atoms [37,38]. The thickness of surface oxidation is 0.5~3 nm under the process of static condition without external disturbance, which prevented the further internal oxidation and provided a functional mechanical skin, approximately charactering from following equation [38,39,40,41],

(1) $$\frac{1}{l}=A-B\ln t$$

where *l* is the thickness of oxidizing film, *A* and *B* are the constants, and *t* is the growth time. However, the gallium oxide that has been generated on the metallic surface could be destroyed by mechanical agitation, so that the oxidation process can continually occur. As shown in Figure 1b, SEM image discloses that the oxidizing liquid metal had the obvious coarse morphology with air hole compared to the glossy surface of the pure liquid metal.

**Figure 2:**
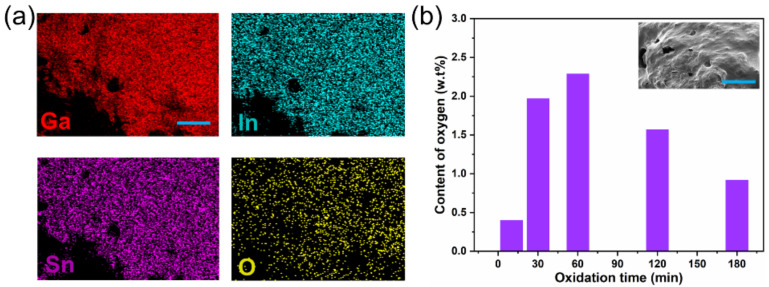
(**a**) The element distribution of oxidizing liquid metal. (**b**) The mass weight of oxygen in different stirring times (scale bar is 100 μm).

**Figure 3:**
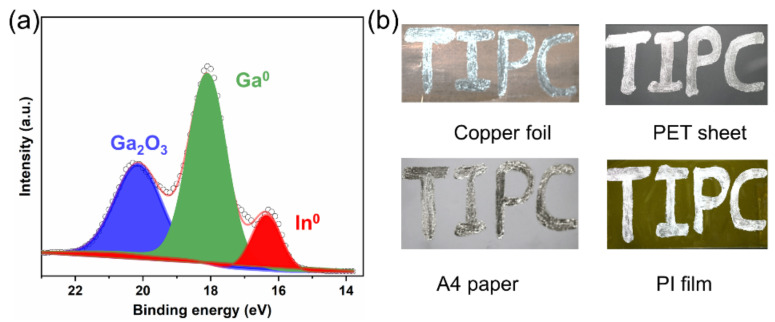
(**a**) The composites and valence states of LM-O_60_ obtained by XPS analysis. (**b**) The printing performance of LM-O_60_ on the diverse substrates.

The distribution of the elemental oxygen was present in Figure 2a, which agrees with that of the metallic elements, only with a lower concentration. This is as oxidation primarily occurred on the metallic surface, and after the surface oxide film was broke, the interior metal then oxidized. Figure 2b displays the mass fraction of oxygen content with different oxidizing times, which revealed that the oxidation degree of the liquid metal increased with the time augmenting. When the stirring time reached 60 min, the oxygen content reached the maximum (2.4 wt.%), indicating that the oxygen molecules have completely penetrated the liquid metal, and the whole structure seems to become a paste-like shape. The corresponding XPS analysis manifested that a considerable amount of oxides generated on the surface are Ga_2_O_3_, in a completely oxidized state (Figure 3a). The printing performance on the surface of flexible substrates is critically decisive to the stable performance of the heating device. The flexibility and universality of the substrates, the printable copper foil, polyethylene terephthalate (PET) sheet, cellulose paper (A4 paper), and PI film sheet are all selected to evaluate the wetting behaviors in sequence, with LM-O_60_ as the printing ink. The experimental results illustrated that the complete oxidation is in favor of the pattern without selectivity of substrates. Then, the PI film was used as the base material of the heating device due to the feature of the ultrathin thickness, wide temperature range, and perfect electrical insulation compared with three other materials. Additionally, LM-O_60_ was the conductive layer, unless specified.

**Figure 4:**
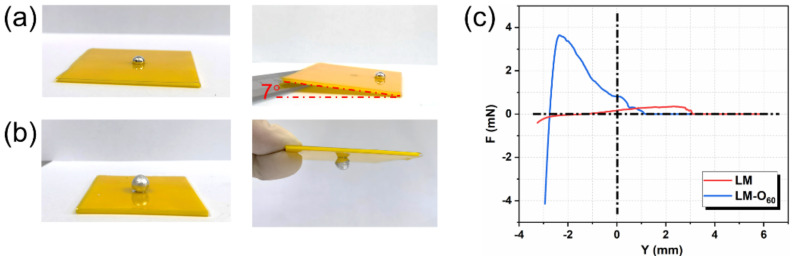
(**a**) The slope experiment of (**a**) pure liquid metal and LM-O_60_ (**b**), the radius of the droplet is 5 mm. (**c**) Force–location (F–Y) curves of LM and LM-O_60_ with same immersion depth (3.5 mm) under the adhesion force measurement.

The adhesion characteristics of liquid metal are the cornerstone for its rapid printing and functional display on the flexible PI film surface. Figure 4a depicts that, in a slope experiment, pure liquid metal would slip at the inclination angle of 7°. However, the LM-O_60_ droplets can maintain the original state on the surface of the flexible film, even if the plane is flipped (Figure 4b). To further explain this phenomenon, the adhesion force testing was performed. As shown in Figure 4c, when the push–pull depth is the same (3.5 mm), the oxidized liquid metal can show significantly stronger adhesion behavior to PI film, indicating that the oxidized liquid metal has better adhesion force than that of pure liquid metal.

### 3.2. The Microstructural Characteristics of the Composite Film

**Figure 5:**
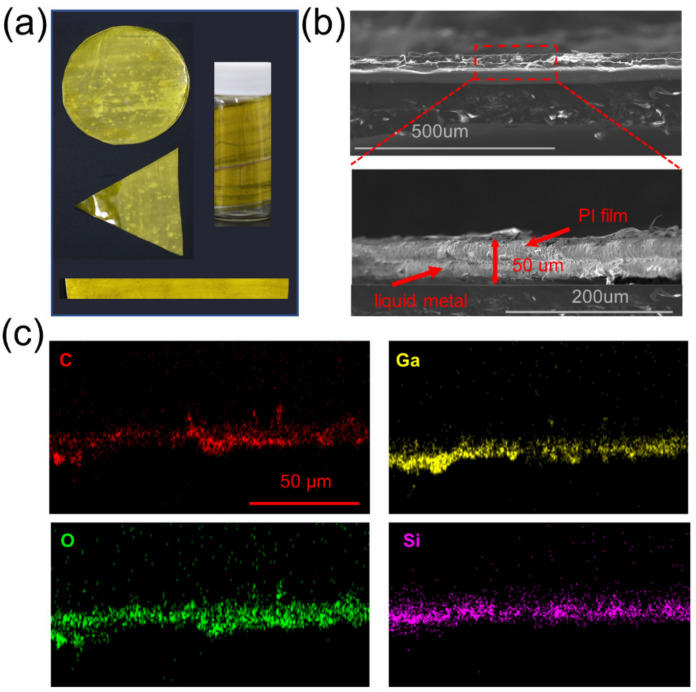
(**a**) Optical images with different shapes of the composite film. (**b**) SEM morphology of the preparing electrothermal film. (**c**) The elemental distribution of the preparing electrothermal film.

**Figure 6:**
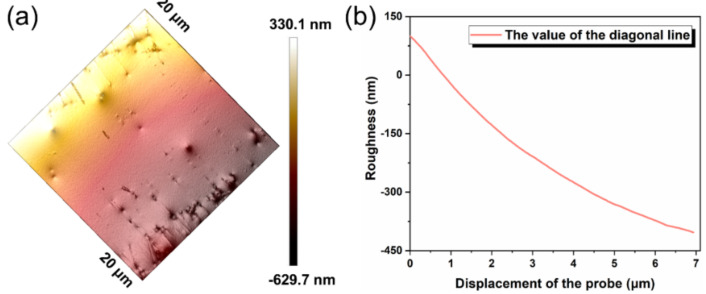
The 3D surface roughness of the composite film. (**a**) The plane roughness recorded by AFM. (**b**) The roughness value along the diagonal line.

Next, the electrothermal film based on the LM-O_60_ conductive layer and PI insulating layer was fabricated via scraper strategy, and Figure 5a presents the optical images of various shapes of the prepared films. The inherent flexibility of the ingredients enabled to be tightly wrapped in a smooth glass wall. The thickness of prepared composite film was about 50 μm, which was composited with two-layer films (Figure 5b), ensuring the connection of the circuit. Additionally, the responding map of EDS confirmed that the elemental oxygen is attaching with the surface of PI film (Si), indicating the oxidization is contributing to the adhesive property of liquid metal. Furthermore, the surface roughness of the prepared film is measured by the AFM with the scan-assist software, which demonstrated that the surface of the prepared film was highly smooth and effectively prevented the circuit breakage caused by the bulging surface structure (<300 nm, in Figure 6).

**Figure 7:**
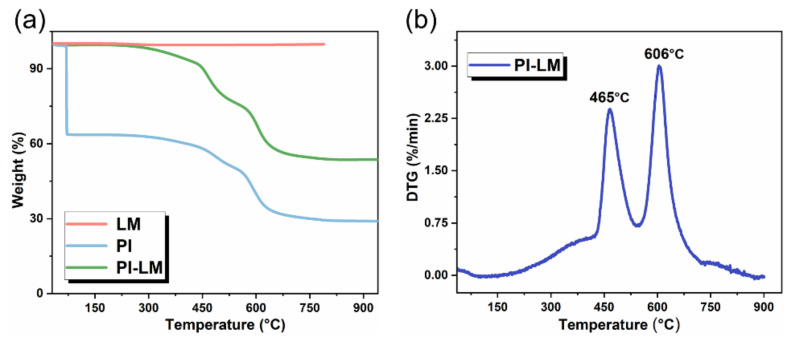
(**a**) The weight change of the prepared PI-LM film. (**b**)The DTG plotting of the prepared PI-LM film.

In order to measure the thermal stability of the PI-LM film, the heat endurance is measured using the TGA testing system, shown in Figure 7. The weight of the LM-PI film coated with LM-O_60_ began to decrease at 300 °C, whose temperature is higher than that of the pure PI film without liquid metal coating (shown in Figure 7a). Additionally, Figure 7b showcases decomposition temperature of the prepared film at two points of 465 °C and 606 °C, corresponding to the melting process and oxidative decomposition process, respectively.

### 3.3. The Electromechanical Performance of the Composite Film

**Figure 8:**
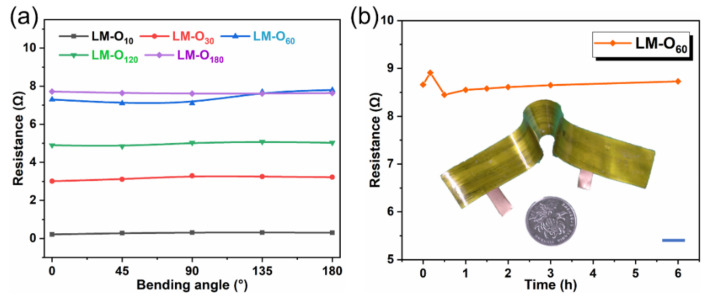
The mechanical and electrical properties of the prepared film. (**a**) The electrical resistance testing when different bending angles. (**b**) The resistance-change with long time testing (the scale bar is 10 mm).

Then, the mechanical and electrical performances of such film were measured. Herein, the copper electrode with decent conductivity was applied to connect and facilitate resistance measurement. Figure 8a exhibits the resistance change of the film with successive angular bending, which demonstrated that the folding behavior has few effects on the conduction of circuits. This is due to the fact the commercial PI film is soft, and the liquid metal conductive layer is firmly adhered to the substrate. For the value of resistance, the resistance of liquid metal increased with the growth of oxidation time. The abnormal resistance of LM-O_120_ may be attributed to the density of oxide layer and the uniformity of infiltration process. The introduction of oxygen hindered the directional motion of electrons and elevated the resistance of the material. However, the resistance would stabilize as the oxidation process is completely conducted. Additionally, to measure the resistance performance over a long working time of the composite film, as shown in Figure 8b, the four-probe method is used to measure the resistance and take the average value of three measurements as the resistance value; the resistance of the film did not change significantly after continuous testing for up to 6 h, indicating excellent electrical stability.

### 3.4. The Electrothermal Performance of the Composite Film

**Figure 9:**
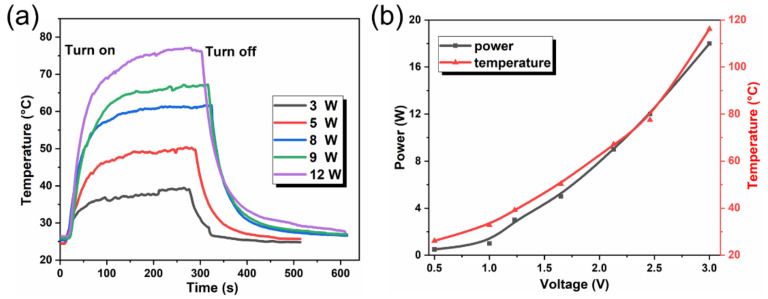
(**a**) The time-dependent temperature curve of PI-LM film as a function of the applied power. (**b**) The plot of temperature and power at the same voltage.

The heating performance of the PI-LM film is displayed in Figure 9a, with the power of electric power ranged from 2 W to 12 W. In the heating process, the film can be divided into three stages: heating stage, heat stabilization stage, and cooling stage. When the current was applied, temperature stability can be achieved within 100 s due to its considerable conductivity and limited heat capacity of the liquid fluids [42]. Additionally, the heating temperature of the circuit raised with the applied power increasing owing to the thermal effects. Moreover, its temperature can return to ambient temperature in a short time (<50 s) as the power is off. This fast temperature switching feature would contribute to potential demands of the rapid heating and cooling, such as the environment requiring accurate high thermal stress.

The curves of the electric field power and temperature of film surface are described in Figure 9b. The results demonstrated that both parameters have a same trend, which is consistent with Joule’s law and Fourier’s law. Moreover, the highest temperature reached 118 °C, while the theoretical temperature of the PI-LM film was 115 °C based on the flowing equation

(2) $$Q=hA\left(T_{0}-T_{m}\right)+\sigma A\left(T_{0}^{4}-T_{m}^{4}\right),$$

where *Q*, *h*, *A*, *σ*, *T*_0_, and *T_m_* are the power, convective heat transfer coefficient, heating area, radiation coefficient, finishing, and ambient temperature, respectively. The agreement between theoretical and experimental results testified the high efficiency of the thermoelectric conversion.

**Figure 10:**
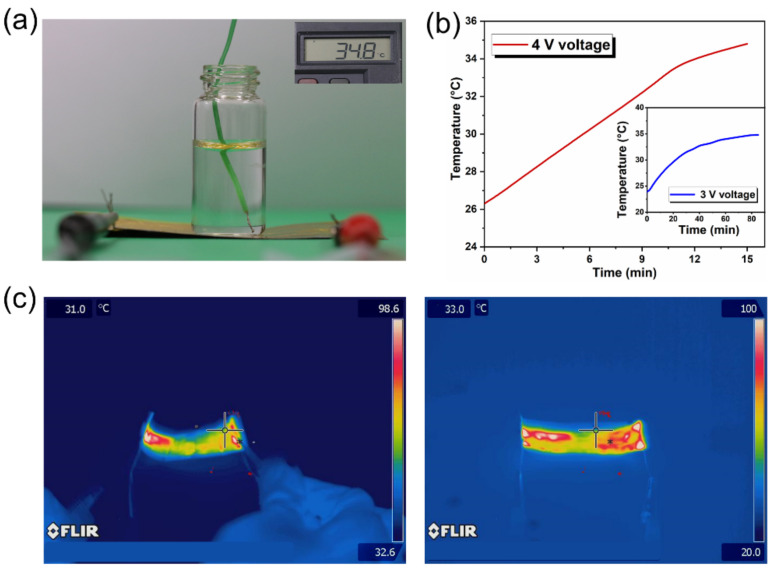
(**a**) The device for the measurement of heating feature. (**b**) Curve of time required to reach different voltages at the same temperature. (**c**) Infrared images of electrothermal feature after liquid nitrogen treatment.

Finally, heating experiments on a bottle of water were conducted to evaluate the electrothermal device. The measuring device is illustrated in Figure 10a,b depicting the time as the function of voltages when the temperature varies from room temperature to 35 °C (10 mL water within 15 min). During the testing process, the time required for the 3 V voltage is six times longer than that of the 4 V. The temperature resistance and mechanical stability for the heating film have a decisive influence on working stages. To measure the stability of the heating film at an extreme cold temperature (−196 °C), the PI-LM film was dropped into liquid nitrogen for 5 min, then it was taken out and subjected to a heating experiment as shown in the infrared image of Figure 10c,d. During the heating process, the film bending generated rare effect on the heating performance, which indicated the flexibility of the film.

## 4. Discussion

It is one of the dominating features of liquid metal with natural thermal and conductive properties. However, the liquid metal is always in a tendency of droplet shrinkage when printing due to the large surface tension [43], and it is necessary to achieve a smoother printing process through the methods of the additives or partial oxidation. The full oxidation technology used here can efficiently realize liquid metal printing without the restriction of substrate, significantly reducing the selectivity of liquid metal printing to substrates, which can be easily written and printed on various surfaces, illustrating a brand new avenue for the universal printing of liquid metal.

On the other hand, this process has a greater cost advantage compared to the previous adhesion method in which metallic particle additives change the wettability. Traditional metallic particles such as nickel, iron, copper, and silver have high prices and potential risks of phase delamination [15,44,45,46]. However, in natural environments, the strategy of controlling the agitation time to achieve viscosity adjustment, economizing the cost of the additives, offered a unique path to reduce the cost of liquid metal printing technology. This low-cost oxidation method to solve the gallium-based liquid metal printing substrate selection characteristics could further strengthen the advantages of low melting point alloys in the field of flexible electronic printing, achieving multifunction and potential applications in detection sensing, flexible robotics, and biomedical engineering.

## 5. Conclusions

In summary, a stable and facile fabrication of composite film by utilizing liquid metal printing for integrated electronics and electrothermal devices has been proposed and demonstrated. The influence of diverse oxidizing time of liquid metal, and the corresponding change in resistance, were discussed. Benefiting from the adjustable adhesive of liquid metal through oxidizing strategy, the liquid metal was enabled via integration on the soft PI film, exhibiting good electromechanical and electrothermal performance, even working normally under extreme conditions. These properties render PI-LM film with potential applications in the field of flexible electronics, personal thermal management, and soft robotics.
